# Prevalence of psychological disorders among caregivers of children with intellectual disabilities and motor disabilities in Shiraz

**DOI:** 10.1192/j.eurpsy.2021.1915

**Published:** 2021-08-13

**Authors:** F. Bakhshisamarin, S. Mirzaei, F. Ansarizangakani, L. Mousavi, A. Sorifijini

**Affiliations:** 1 Psychiatry, hospital, Tehran, Iran; 2 Psychology, sanandaj University, sanandaj, Iran; 3 Psychology, orumieh University, orumeih, Iran; 4 Psychology, azad University, shiraz, Iran; 5 Psychology, arak University, arak, Iran

**Keywords:** Prevalence, psychological disorders, intellectual disabilities, motor disabilities

## Abstract

**Introduction:**

The prevalence of mental disorders plays an important role in identifying the state of mental health of the community and estimate the required facilities at any time.

**Objectives:**

Therefore, the purpose of this study was to investigate the prevalence of psychological disorders in caregivers of children with intellectual disabilities and motor disabilities in Shiraz.

**Methods:**

The research was descriptive and cross-sectional survey. The statistical population consisted of all mothers of children with disabilities who were admitted to welfare centers and clinics of Shiraz in 2019. Of these families, 35 mothers with children with intellectual disability and 35 mothers with children with motor disability were selected as the sample group. SCL-90-R (1976) was used to collect of data. For analyze the data, MANOVA test was used.

**Results:**

The results of this study showed that the most common psychological disorders in caregivers of children with intellectual disability were aggression (hostility), hypersensitivity to interpersonal relationships, anxiety and depression, and in caregivers of children with motor disabilities were physical complaints, sensitivity to interpersonal relationships, paranoid thoughts and anxiety.

**Conclusions:**

According to the findings of this research, it can be concluded that caregivers of children with special needs in terms of mental health are not in a favorable situation, which is due to the lack of proper adaptation with the disability of their child and the failure to meet their needs.

**Disclosure:**

No significant relationships.

